# Experimental Characterization of Close-Emitter Interference in an Optical Camera Communication System

**DOI:** 10.3390/s17071561

**Published:** 2017-07-04

**Authors:** Patricia Chavez-Burbano, Victor Guerra, Jose Rabadan, Dionisio Rodríguez-Esparragón, Rafael Perez-Jimenez

**Affiliations:** 1 Facultad de Ingeniería en Electricidad y Computación, Escuela Superior Politécnica del Litoral (ESPOL), Campus Gustavo Galindo Km 30.5 Vía Perimetral, P.O. Box 09-01-5863 Guayaquil, Ecuador; 2 Institute for Technological Development and Innovation in Communications (IDeTIC), ULPG, Las Palmas 35001, Spain; vguerra@idetic.eu (V.G.); jrabadan@idetic.eu (J.R.); rperez@idetic.eu (R.P.-J.); 3 Instituto de Oceanografía y Cambio Global (IOCAG), ULPG, Las Palmas 35214, Spain; dionisio.rodriguez@ulpgc.es

**Keywords:** Optical Camera Communication (OCC), Interference, Normalized Power Signal to Interference Ratio (NPSIR), Wireless Sensor Networks (WSN)

## Abstract

Due to the massive insertion of embedded cameras in a wide variety of devices and the generalized use of LED lamps, Optical Camera Communication (OCC) has been proposed as a practical solution for future Internet of Things (IoT) and smart cities applications. Influence of mobility, weather conditions, solar radiation interference, and external light sources over Visible Light Communication (VLC) schemes have been addressed in previous works. Some authors have studied the spatial intersymbol interference from close emitters within an OCC system; however, it has not been characterized or measured in function of the different transmitted wavelengths. In this work, this interference has been experimentally characterized and the Normalized Power Signal to Interference Ratio (NPSIR) for easily determining the interference in other implementations, independently of the selected system devices, has been also proposed. A set of experiments in a darkroom, working with RGB multi-LED transmitters and a general purpose camera, were performed in order to obtain the NPSIR values and to validate the deduced equations for 2D pixel representation of real distances. These parameters were used in the simulation of a wireless sensor network scenario in a small office, where the Bit Error Rate (BER) of the communication link was calculated. The experiments show that the interference of other close emitters in terms of the distance and the used wavelength can be easily determined with the NPSIR. Finally, the simulation validates the applicability of the deduced equations for scaling the initial results into real scenarios.

## 1. Introduction

Nowadays, there is a trend in replacing fluorescent and halogen lamps, both indoor and outdoor, by LED-based ones, which are energy efficient and offer extended life cycles and high switching speed. This change also affects street, traffic and car lights (hazard, tail, sidelights, and so forth), opening the opportunity of deploying new communication systems over the fundaments of Optical Camera Communications (OCC) [1] for a wide range of applications, e.g., in smart cities or V2X networks. OCC is a specific kind of VLC-based technique [2] that uses LED-based devices (such as lamps, or screens) for transmitting data, and image sensors for receiving the signals. OCC deployment is low cost, offers immunity to Radio Frequency (RF) interference, and takes advantage of the fast insertion of devices with embedded cameras in day-to-day activities. VLC techniques, including OCC, solve critical issues of the current RF telecommunication implementations: the spectrum saturation and the interferences from widespread RF systems.

Due to the use of visible light signals and photoreceivers, there are other interfering sources that should be taken into account during channel characterization and link design stages. Weather conditions, the possible image distortions due to both optical turbulence [3,4] and lens aberration [5], the solar radiation interference, and other light sources constitute the main problems that outdoor systems need to consider. Some research works modeling the impact of climate conditions on the system’s performance have been published. In this sense, the effect of snowfall was simulated in [6], showing that the attenuation and the time variation of the received signal cannot be ignored and depends on the size distribution of the snowflakes due to Fresnel diffraction. In [7], the influence of rainfall in VLC links was analyzed. This work established that the optical transmitted signal is scattered when hits a raindrop, so the received power diminished according to the rain rate (number and size of raindrops). In addition, the attenuation introduced by fog in Free-space optical (FSO) communications was studied in [8], showing that it can be predicted according to visibility scenarios without using heavy computer codes. These channel modeling research works were based on photodiode-based receivers.

Nevertheless, in an attempt of characterizing multi-element receivers, such as cameras, Ashok et al. [9] studied the effect of distance using a photodiode array and defined the critical distance where the LED generates an image of one pixel only. Moreover, the viewing-angle dependency was characterized in [10] in terms of the Signal-to-Noise ratio (SNR). The study of self-interference in OCC as a performance degradation factor has been addressed in [10,11]. However, the chromatic effect has not been discussed. In this topic, the characterization of vehicle-to-traffic-light communications assuming that the link is affected only by background solar radiation (for yellow, red and green) and artificial light sources (lighting and advertising) excluding close emitters from the same system was presented in [12]. Some practical issues related to the use of multi-LED transmitters with chromatic modulation were addressed by [13] for OCC, but only the viewing angle distortion was used as a critical parameter. Finally, Hong and Chen [14] presented the effect of mobility on the performance of these systems using photodiodes as receivers. It was shown that the system performance depends on the speed; if the users move faster, the packet loss increases.

The application of the Internet of Things (IoT) over a heterogeneous network has become a current trend [15]. This novel network ecosystem uses a large variety of transmission data rate. For slow data rate applications, such as in wireless sensor networks (WSN) which require transferring minimum amounts of data (for example temperature, pressure, humidity, or presence flags), the use of preexisting cameras-based infrastructure is a possible solution. This use of cameras as information gathering devices, not only decreases the infrastructure inversion, but it also increases the system security. If the signal has been intercepted, an additional signal processing will be required in order to extract the relevant information. Additionally, for security cameras, such as from CCTV systems, the communication is already protected by design. Moreover, the use of OCC in WSN allows the implementation of Multiple Input Multiple Output (MIMO) systems as well as Space Division Multiple Access (SDMA) solutions [16,17].

**Figure 1:**
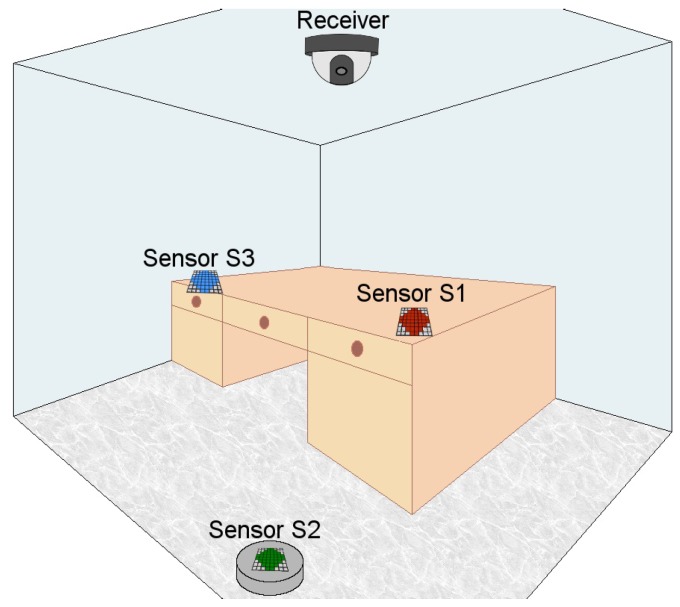
Wireless Sensor Network implementation using Optical Camera Communication (OCC) in an office (3 × 3 × 3 m^3^). The receiver is a dome camera installed in the ceiling (height 3.00 m) with 90° Field of View (FOV). The sensor’s emitters are LED matrices (60 × 60 mm^2^). Two sensors are located on the desk (height 0.80 m) and one sensor is over an autonomous robotic vacuum cleaner (height 0.10 m).

In this work, the interference that another emitter close by the particular transmitter of an OCC system can introduce into the communication link is experimentally characterized. Specifically, working with RGB multi-LED transmitters, such as LED matrices, and commercial middle range cost cameras. For this purpose, the Normalized Power Signal to Interference Ratio (NPSIR) is presented in order to measure the chromatic interference independently of the chosen camera or the optical power transmitted by the LEDs. Some functions for easily determining the 2D pixel representation of real separation distances are deduced and validated. These functions are also used for projecting proportional distances into a real scenario, as shown in Figure 1, where a WSN is deployed in a small office with three sensors transmitting in different wavelengths using OCC with On-Off Keying modulation (OOK). Finally, the corresponding Signal to Interference Ratio (SIR) of the sensors over each other is determined in function of the NPSIR obtained from the experimental characterization.

This paper is organized as follows: in Section 2 the fundamental theory is presented; the materials and methods of the experiments are detailed in Section 3 and Section 4 present the obtained results. A real case scenario simulation is described in Section 5 along with the corresponding results. The discussion, conclusions, future works and possible applications are presented in Section 6.

## 2. Theoretical Fundaments

Based on trigonometric relations and camera parameters, Equations (1) and (2) have been obtained. These equations estimate the 2D pixel projection (*x*, *y*) in a picture of a real distance ($d_{x}$, $d_{y}$, *D*) in function of the separation between the object and the camera, the relative angle of the object (vertical or horizontal), and the camera’s specific characteristics.

(1) $$x=\frac{1}{\varphi_{x}}\cdot \left[\left(tan^{-1}\frac{d_{x}sin\gamma }{2D-d_{x}cos\gamma }\right)+\left(tan^{-1}\frac{d_{x}sin\gamma }{2D+d_{x}cos\gamma }\right)\right]$$

(2) $$y=\left\{\begin{array}{ll} \frac{2}{\varphi_{y}}\cdot \left[tan^{-1}\frac{d_{y}}{2D+d_{x}cos\gamma }\right] & ;\;\mathrm{Farthest elements}\\ \frac{2}{\varphi_{y}}\cdot \left[tan^{-1}\frac{d_{y}}{2D-d_{x}cos\gamma }\right] & ;\;\mathrm{Closest elements} \end{array}\right.$$

**Figure 2:**
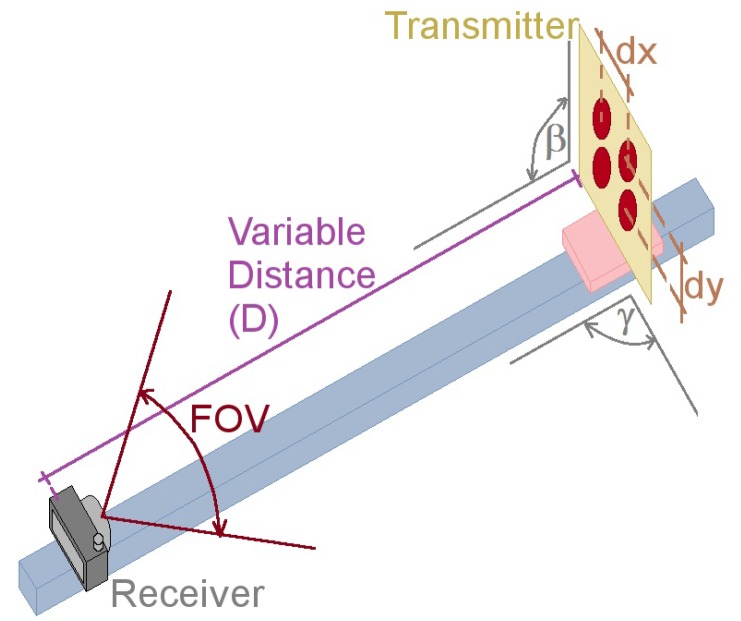
Experimental testbed design for determining the optical interference of close emitters and for validating the relation between projected pixels and real distances. For all the experiments β was set to 90°, γ and *D* varied according to the test parameters.

As shown in Figure 2, *D* is the distance between transmitter and receiver, $d_{x}$ and $d_{y}$ are the horizontal and vertical distances between the sources respectively, γ is the angle of the emitter-receiver vertical plane intersecting the emitter’s normal plane, and β is the angle of the emitter-receiver horizontal plane intersecting the transmitter’s normal plane. Finally, φ depends only on the receiver characteristics and relates the Field of View (FOV) and the resolution in pixels ($N_{x,y}$) of the selected camera.

**Figure 3:**
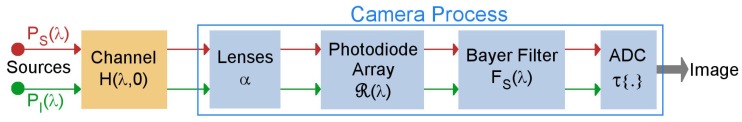
OCC System Diagram.

For determining the optical interference, the relation between the pixels from the link transmission source and the pixels from one close emitter within the corresponding channel (related with the selected wavelength) was deduced. The communication system has two main blocks: the channel and the camera process, as can be observed in Figure 3, where P(λ) is the transmitted optical power spectrum; H(λ,0) is the channel gain; α is a geometric correction factor for each light ray depending on how it fell on the lenses; R(λ) is the photodiode Silicon Responsivity; $F_{S}\left(\lambda \right)$ is the camera’s Bayer Filter response over the channel of the source S; τ is a nonlinear transformation due to the possible saturation from the analog/digital converter; and the image is the formed frame including all the sources.

The light from any source X that arrives at the camera can be expressed as $\int P_{X}\left(\lambda \right)H\left(\lambda ,0\right)d\lambda$. First, the lenses will focus the light into the image sensor where each device has a Silicon Responsivity R(λ). Depending on the selected channel (Red, Green or Blue), the camera will use a Bayer Filter with response $F_{S}\left(\lambda \right)$ over the signal. Finally, the analog-to-digital converter (ADC) transforms the signal using the function τ{·}. Therefore, in the obtained image the amount of pixels from a source X can be expressed by $\alpha_{X}\cdot \tau \left\{\int P_{X}\left(\lambda \right)H\left(\lambda ,0\right)R\left(\lambda \right)F_{S}\left(\lambda \right)d\lambda \right\}$.

This Signal to Interference and Noise Ratio (SINR) depends only on the amount of pixels in the frame with information from multiple sources simultaneously. For this work, the worst case scenario is assumed, both light sources are transmitting simultaneously all the time. Obviously, since the camera’s shutter speed defines the integration time of the image, and the two LEDs are turned on synchronously, the SINR is not affected by the camera’s shutter speed. The deduced pixel relation can be observed in (3), where $\alpha_{S}/\alpha_{I}$ is the geometric ratio, which is the relation between the number of pixels of signal respect to the number of pixels of interference within the frame’s region of interest (ROI).

(3) $$\frac{Pix_{S}}{Pix_{I}}=\frac{\alpha_{S}}{\alpha_{I}}\cdot \frac{\tau \left\{\int P_{S}\left(\lambda \right)H\left(\lambda ,0\right)R\left(\lambda \right)F_{S}\left(\lambda \right)d\lambda \right\}}{\tau \left\{\int P_{I}\left(\lambda \right)H\left(\lambda ,0\right)R\left(\lambda \right)F_{S}\left(\lambda \right)d\lambda \right\}}$$

Since the distance variation between emitters and the receiver is minimal, the geometric related part of H(λ,0) can be considered dependent only of *D*. Additionally, the scenario is LOS (Line of Sight) with short distances without aerosols, so the extinction factor is unitary for all λ. Therefore the term H(λ,0) can be considered as constant and can be taken out of the integral and simplified. The Geometric Ratio $\alpha_{S}/\alpha_{I}$, expressed as the Normalized Power Signal to Interference Ratio (NPSIR) can be determined using (4). This interference ratio is independent of the transmitted optical power, the Silicon responsivity, and the camera’s Bayer filter; and can be used as a basic measure for simulating more complex scenarios.

(4) $$NPSIR≅\frac{Pix_{S}}{Pix_{I}}\cdot \frac{\int P_{I}\left(\lambda \right)R\left(\lambda \right)F_{S}\left(\lambda \right)d\lambda }{\int P_{S}\left(\lambda \right)R\left(\lambda \right)F_{S}\left(\lambda \right)d\lambda }$$

Finally, for the simulation of the WSN of Figure 1, the thermal noise can be neglected due to its minimal effect over cameras in environments with controlled temperature conditions. The ambient light is assumed to be set as a low power intensity signal, constant over the transmission time. Therefore the interference of nearby transmitters can be considered the only source that will significantly affect the communication link. Additionally, since the selected dome camera for the WSN scenario has a CCD image sensor, the shutter type is assumed as global.

## 3. Experimental Design

**Figure 4:**
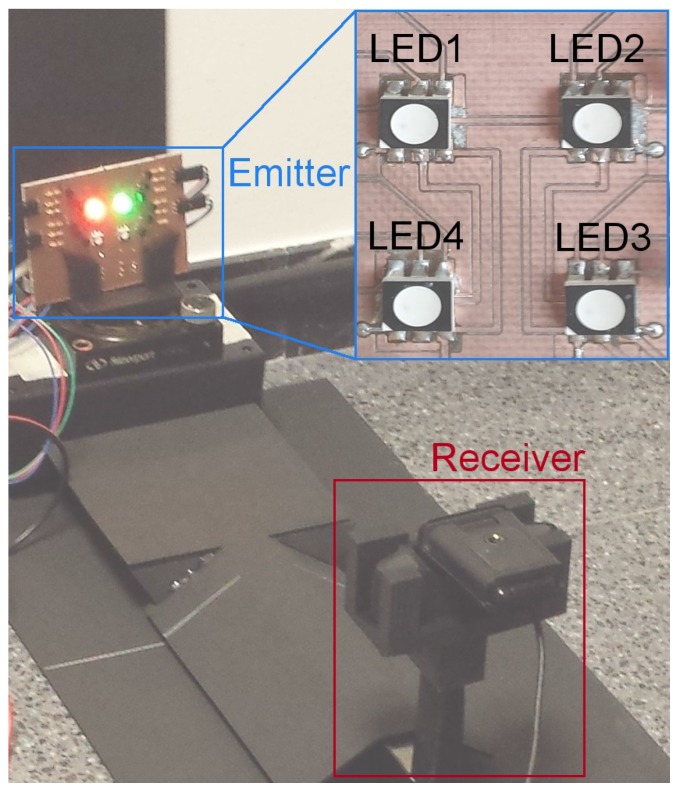
Implemented experimental testbed. The emitter is the designed board, controlled externally. In this case, the top LEDs were turned on in red (LED1) and green (LED2). The receiver, a USB webcam, was controlled by a PC and was located over a rail at 40 cm from the transmitter.

In order to validate the proposed Equations (1) and (2), and to determine the optical interference of close emitters within an OCC system, in function of the transmitters’ location, the selected data transmission wavelength and the distance between the system’s elements, a set of test was performed using the testbed of Figure 4, which was designed and implemented specifically for these experiments. 

**Figure 5:**
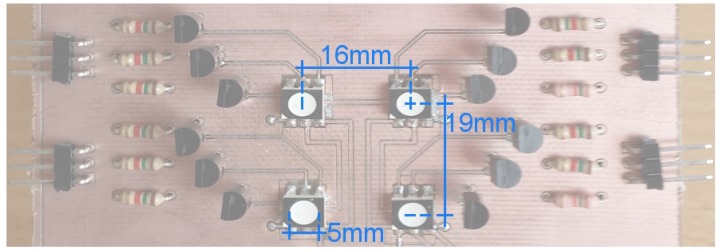
Designed LED’s Board with the distances between its elements.

The board shown in Figure 5 was used as the emitter; the four RGB LEDs were circular (radius of 5 mm) with viewing angle of 120°, separated horizontally 16 mm and vertically 19 mm. These LEDs were switched ON/OFF using bipolar transistors and a small single board computer programmed with Python. In order to have all possible combinations, each LED was controlled independently, changing its colors with four possible outcomes: red, green, blue and white.

The receiver was a commercial USB webcam with 640 × 480 pixels resolution and diagonal FOV of 78°, which was controlled by a script for capturing frames with all the possible interference combinations, so the same parameters of image capture were assured. The camera was located on a rail in order to guarantee the alignment with the transmitter when the distance between the elements varied.

### 3.1. Equation Validation

In pursuance of validating the 2D pixel representation equations, the following experiment was performed. The angle β was set to 90°, whilst γ was set to four values: 45°, 60°, 75° and 90°. The distance *D* varied from 20 cm to 200 cm stepped off 20 cm. At each position (different distance and angle) 40 pictures were taken with different color combinations for the turned on LEDs. The acquired photos were processed for determining the location of the light sources, their geometric shape (in our particular case, the form should match a disc) and their centroids’ coordinates. With this information, the experimental distance between the LEDs (horizontal and vertical) was calculated and compared with the theoretical ones obtained from Equations (1) and (2). The horizontal mean error was 0.91777 pixels and the vertical mean error 0.86238 pixels (0.91070 pixels for the set of far LEDs, and 0.81119 pixels for the set of close LEDs). Since in both cases the mean error was less than one pixel, we assumed that the equations were validated.

### 3.2. Optical Interference

**Figure 6:**
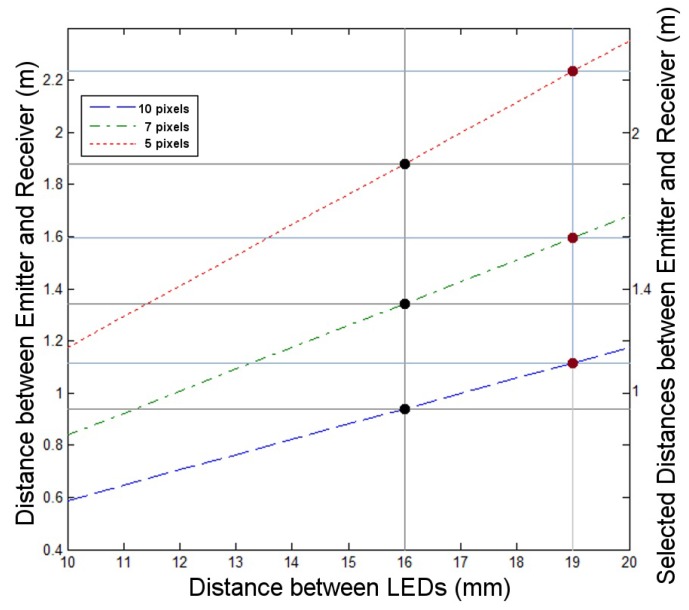
Distance between emitter and receiver for achieving a specific number of pixels (10, 7 and 5 pixels) in function of the LED separation. The distances of 0.016 m (black dots) and 0.019 m (red dots) represent the horizontal and vertical separation of the RGB LEDs in the testbed board respectively. The values in the right were the selected ones for each specific pixel number.

For determining the optical interference of close emitters within an OCC system an experiment was developed. The angles β and γ were set to 90°, while the distance *D* (between the LED board and the camera) varied. Adjusting the distance *D* the test could have three scenarios: a perfect spatial separation between the LEDs among the pictures (pixel distance greater than 10); a limited spatial separation (pixel distance between 6 and 9); and a critical spatial separation (pixel distance less than 5). These link ranges were deduced using Equations (1) and (2), previously validated, with the particular parameters of this experiment, as shown in Figure 6.

- Distance for perfect spatial separation: 100 cm.
- Distance for limited spatial separation: 140 cm.
- Distance for critical spatial separation: 200 cm.

**Figure 7:**
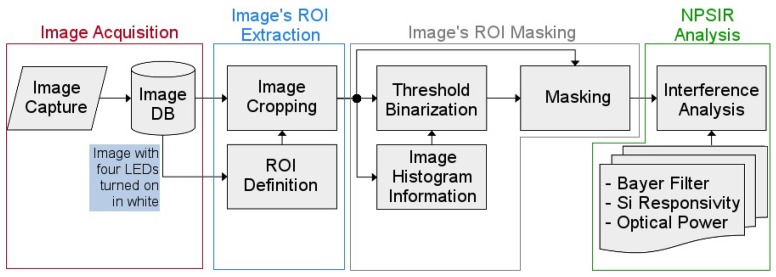
Optical Interference Experiment Methodology Diagram.

As shown in Figure 7, the experiment had four phases: Image Acquisition; Image’s Region of Interest (ROI) extraction; Image’s ROI Masking; and NPSIR Analysis. For this purpose, the board was programmed to turn on the LEDs for each color: blue, green, red and white (the three orthogonal colors at the same time) following the sequence:

- Only one LED at the time (four combinations).
- Two horizontal LEDs at the time (two combinations).
- Two vertical LEDs at the time (two combinations).
- Two diagonal LEDs at the time (two combinations).
- Four LEDs at the time (one combination).

### 3.3. Image Acquisition

During the first stage, images of the programmed sequence were captured in a dark room in order to eliminate interference from other light sources. The camera was set up with maximum contrast (255), no brightness (zero), manual average white balance (4000) and focus to infinity (zero). A script was used for acquiring and storing the 44 pictures automatically at each position.

### 3.4. Image’s ROI Extraction

**Figure 8:**
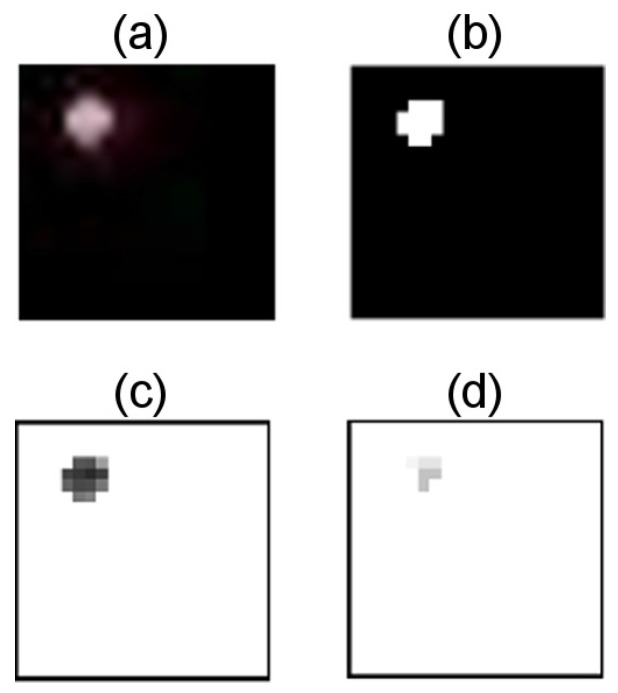
Cropped Image Process. (**a**) ROI of the image with LED1 turned on in red color; the distance between emitter and receiver was set to 100 cm; (**b**) Binary mask obtained with threshold 0.3431; (**c**) Non-zero pixels of the emitting LED’s image, obtained from applying the mask to channel R of the image; (**d**) Non-zero pixels of the interference signal from LED2 turned on in white color, obtained from applying the mask to channel R of the interference image.

For each selected distance, the all-white LEDs image was inspected, looking for the specific rows and columns that delimited the area with the 95% of the pixel value for each dimension. This information was used to automatically extract the specific portion that allocated the LEDs, for all the images. For example, Figure 8a shows the cropped ROI from the image where LED1 was turned on in red color and the distance D was set to 100 cm.

### 3.5. Image’s ROI Masking

**Figure 9:**
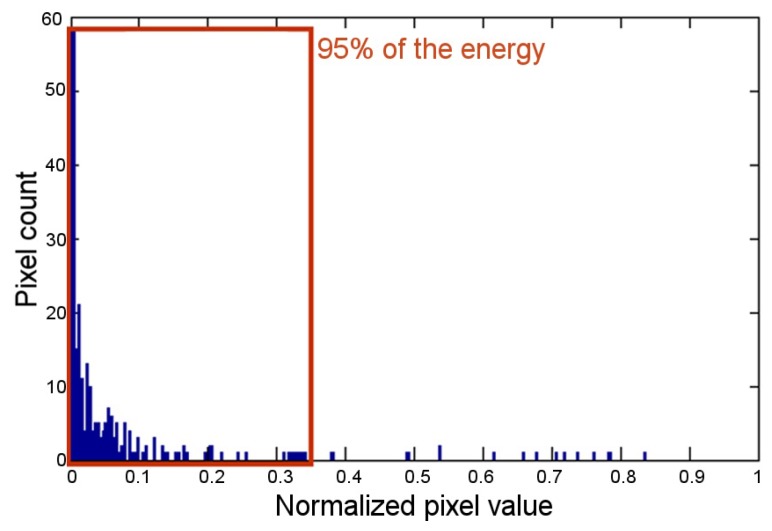
Histogram of channel R of the cropped image with LED1 turned on in red color; the distance between emitter and receiver was set to 100 cm.

Then, this fragment of the original image was processed in order to obtain a binary mask similar to the image in Figure 8b. For this purpose, a dynamic threshold, that assured the conservation of 95% of the energy, was calculated from the corresponding image’s histogram in the specific channel (R, G, or B) of the emitter. The Figure 9 shows the histogram of the previous example image over channel R, where the threshold was measured as 0.3431. Finally, the pixels from the transmitting LED ($Pix_{S}$) were determined by applying the obtained mask to the appropriate channel of the cropped image that contains the emitter LED (see Figure 8c). In the same way, the pixels from the close by interference LED ($Pix_{I}$) were calculated by masking the image that contains the next horizontal or vertical LED (Figure 8d).

### 3.6. NPSIR Analysis

**Figure 10:**
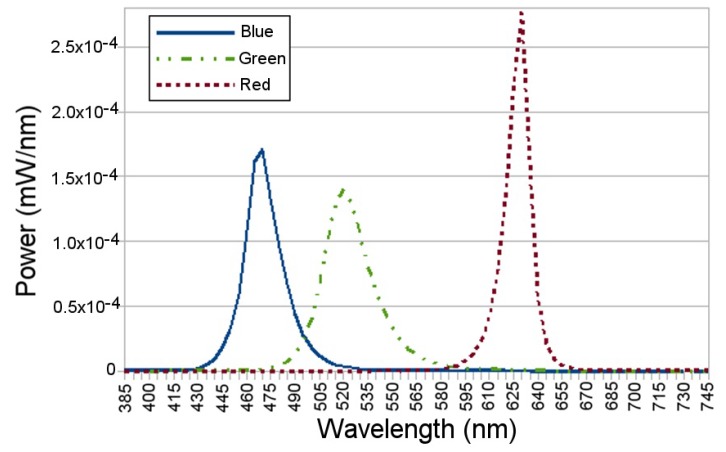
Optical Power emitted by the top left LED of the designed board turned ON in Blue, Green and Red respectively, data obtained using an optical spectrum analyzer.

**Figure 11:**
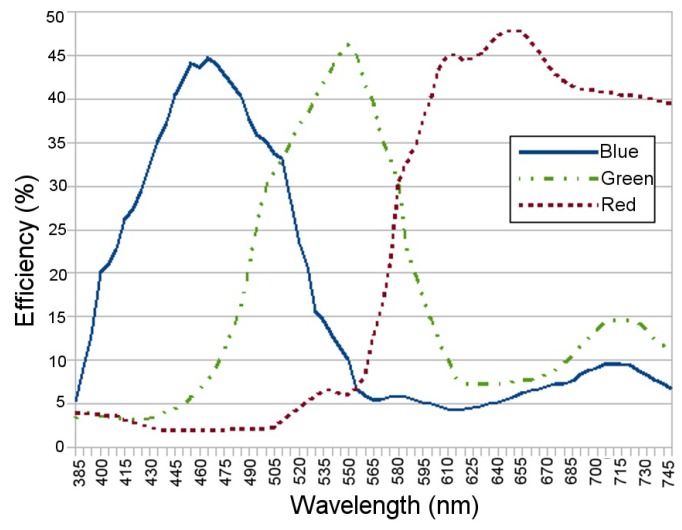
Webcam Bayer Filter Response for Blue, Green and Red respectively data extracted from [18].

**Figure 12:**
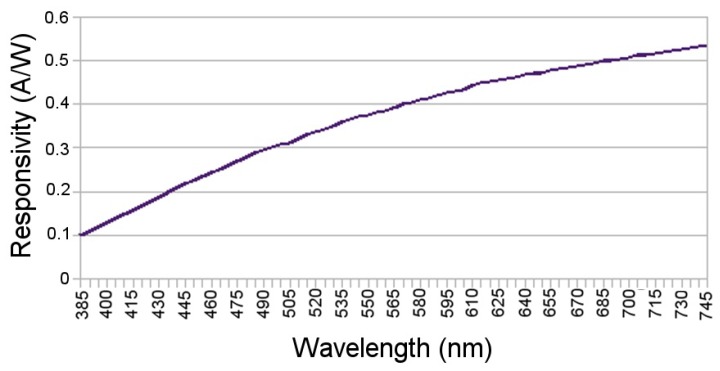
Silicon Responsivity data extracted from [18].

In order to calculate the NPSIR, as shown in Equation (4), pixel count was executed in conjunction with the optical power, the Silicon responsivity, and the Bayer Filter response. Using an optical spectrum analyzer, the optical power emitted by each LED of the board in each color was obtained; Figure 10 shows the particular case of LED1 (top left in Figure 4) turned on in the different colors. Theoretical Bayer Filter response (Figure 11) and silicon responsivity (Figure 12) of the selected camera were obtained from the sensor data sheet [18,19]. Since the data of the optical power were determined by sampling wavelengths from 385 nm to 745 nm with steps of 5 nm; the NPSIR was calculated using a numerical integration of Equation (4), yielding as shown in Equation (5).

(5) $$NPSIR_{dB}=20\cdot log\left[\frac{Pix_{S}}{Pix_{I}}\cdot \frac{\sum_{\lambda =385}^{745}\left(P_{I}\left(\lambda \right)R\left(\lambda \right)F_{S}\left(\lambda \right)\right)}{\sum_{\lambda =385}^{745}\left(P_{S}\left(\lambda \right)R\left(\lambda \right)F_{S}\left(\lambda \right)\right)}\right]$$

## 4. Experimental Results

**Figure 13:**
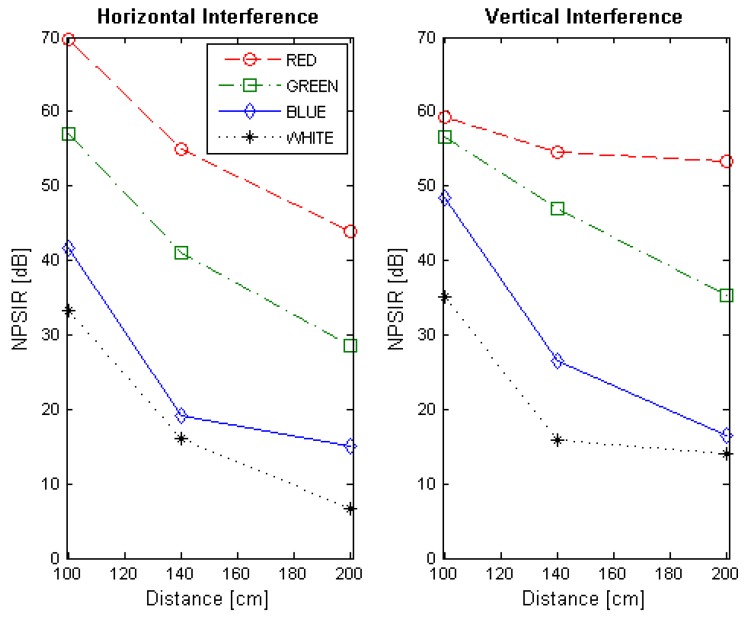
Normalized Power Signal to Interference Ratio for transmitting in blue. The left chart presents the interference of close emitters located on the same horizontal line. Right chart shows the interference of the transmitters within the same vertical line.

**Figure 14:**
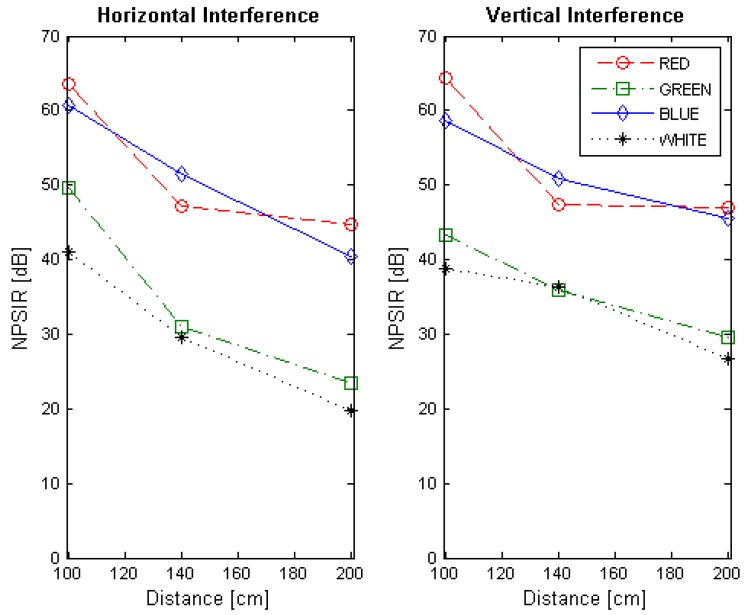
Normalized Power Signal to Interference Ratio for transmitting in green. The left chart presents the interference of close emitters located on the same horizontal line. Right chart shows the interference of the transmitters within the same vertical line.

**Figure 15:**
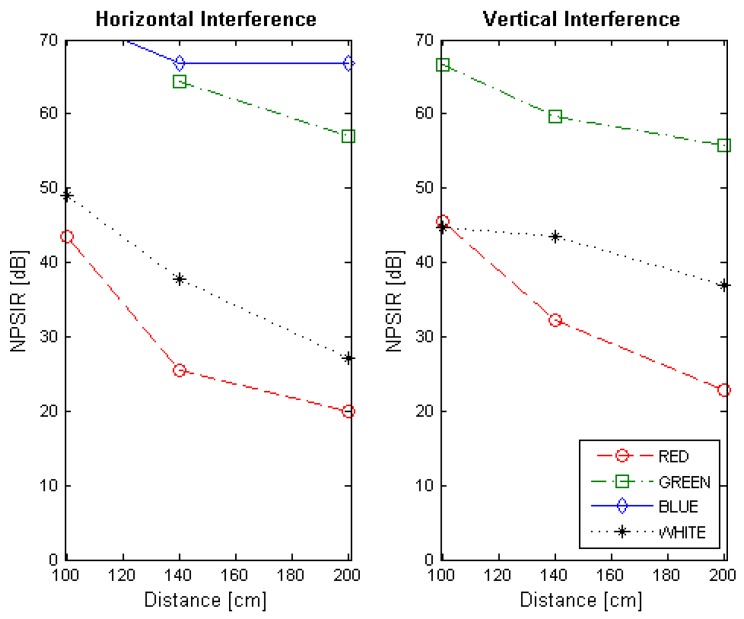
Normalized Power Signal to Interference Ratio for transmitting in red. The left chart presents the interference of close emitters located on the same horizontal line. Right chart shows the interference of the transmitters within the same vertical line.

**Figure 16:**
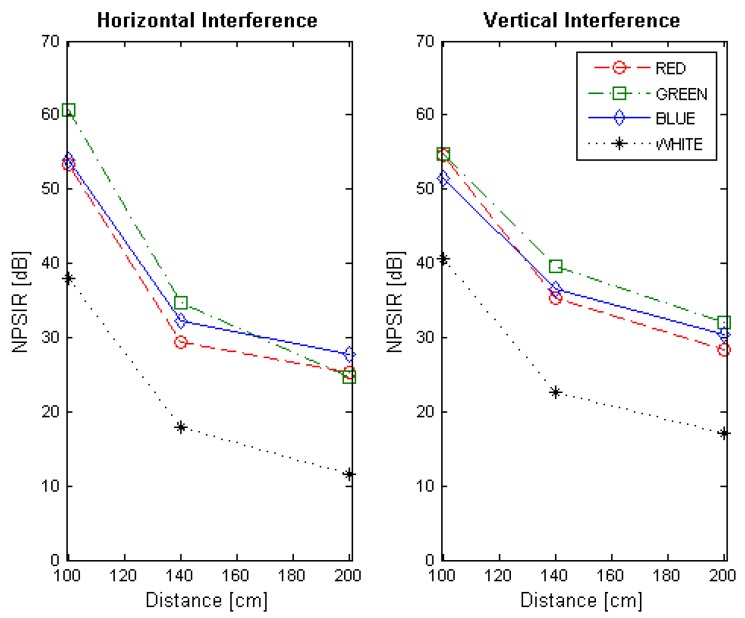
Normalized Power Signal to Interference Ratio for transmitting in white. The left chart presents the interference of close emitters located on the same horizontal line. Right chart shows the interference of the transmitters within the same vertical line.

At the end of the optical interference experiment, the NPSIR for each selected distance, color combination and LED position was calculated. Figure 13, Figure 14, Figure 15 and Figure 16 shows the obtained results transmitting in Blue, Green, Red, and White respectively, in function of the distance between transmitter and receiver. In all the figures, the left plot corresponds to the interference from a LED on the same horizontal line of the transmitter, whilst the right chart presents the interference from a source in the same vertical line. For example, if the emitter is LED1, the horizontal interference came from LED2 and LED4 contributed to the vertical one (see Figure 4). The line color represents the wavelength of the interference, except for the black line that shows the interference from the LED turned on in white.

## 5. Wireless Sensor Network Simulations

**Figure 17:**
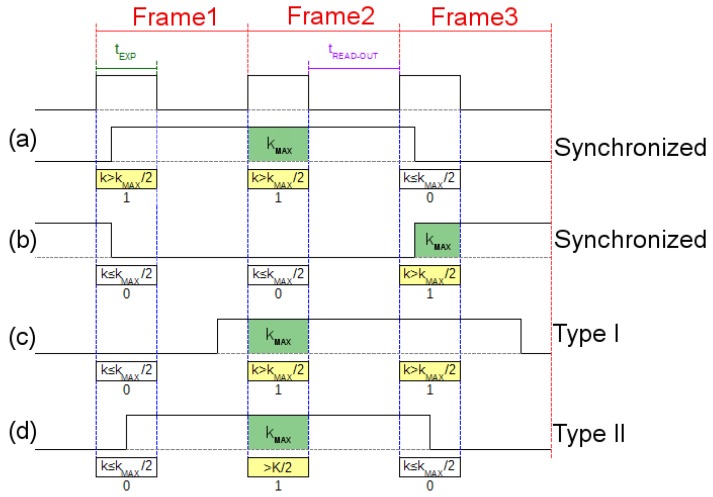
Synchronizing procedure. (**a**) The average pixel value of the second frame is $k_{max}$, therefore the first two frames are decoded as “1” ($k>k_{max}/2$) and the third is decoded as “0” ($k<k_{max}/2$), so the signal is synchronized with the camera and no process is needed. (**b**) The average pixel value of the third frame is $k_{max}$, therefore the first two frames are decoded as “0” ($k<k_{max}/2$) and the third is decoded as “1” ($k>k_{max}/2$), so the signal is synchronized with the camera as in case (a). (**c**) The average pixel value of the second frame is $k_{max}$, therefore the first frame is decoded as “0” ($k<k_{max}/2$) and the other ones are decoded as “1” ($k>k_{max}/2$), so the signal is not synchronized with the camera and type I process is needed; the first frame is discarded in order to synchronize the signal. (**d**) The average pixel value of the second frame is $k_{max}$, therefore the first and third frames are decoded as “0” ($k<k_{max}/2$) and the second is decoded as “1” ($k>k_{max}/2$), so the signal is not synchronized with the camera and type II process is needed; the emitter restart the transmission.

Using the scenario of Figure 1, a simulation was created. The small office was designed as a windowless 3.00 m length × 3.00 m height × 3.00 m width room in order to avoid external light interference. A global shutter dome camera with diagonal FOV of 90° and resolution of 640 × 480 pixels, installed on the ceiling, was selected as the receiver; this camera was set up with the same parameters of the webcam used in the previous experiment (30 fps, focus = 0, contrast = 255, brightness = 0, and white balance = 4000) adjusting the exposure time to 10ms. Three sensors were allocated in the office, each one with an optical emitter, represented by an 8 × 8 RGB LED matrix (6.00 cm length × 6.00 cm width × 0.40 cm height) with viewing angle of 30°. One sensor (S3) was over an autonomous vacuuming Robot (9.10 cm height) and transmitted in Green (528 nm). The other two sensors were over a desk (0.80 m height), S1 emitted in Red (623 nm) and S2 in Blue (467 nm). Each matrix turned on 40 LEDs forming a circle that was assumed as a unique LED with optical power equivalent to the summation of all the elements contribution. The selected modulation for this scenario was OOK-NRZ at 15 bps (each bit sampled in two consecutive frames) without undersampling. For this particular simulation, the matrices are not used for illumination purposes and the flickering effect was of no concern. However, for other scenarios where the flickering is considered bothersome, an undersampling technique should be used to avoid this effect. In order to synchronize the received signal, an adaptation of the frame selection technique proposed in [20] was applied for the three first frames of the transmission. The initial header of the message has a known sequence (101010), so the selected frames have at least one “bit 1”, which has the maximum average pixel intensity value ($k_{max}$). For each frame a bit value is determined, if the average pixel intensity value (*k*) is greater than $k_{max}/2$, the bit is marked as “1”; otherwise, the bit is marked as “0”. The signal is considered synchronized when the two first bits are equal and the third one different (Figure 17a,b); otherwise, the signal is unsynchronized with two possible cases. The first bit is different, but the last two bits are equal (Figure 17c), for this case, the first frame is dropped and the remaining ones are considered synchronized. The first and the last bits are equal, while the middle one is different (Figure 17d), in this case, the signal cannot be used and the emitter needs to restart the transmission.

**Figure 18:**
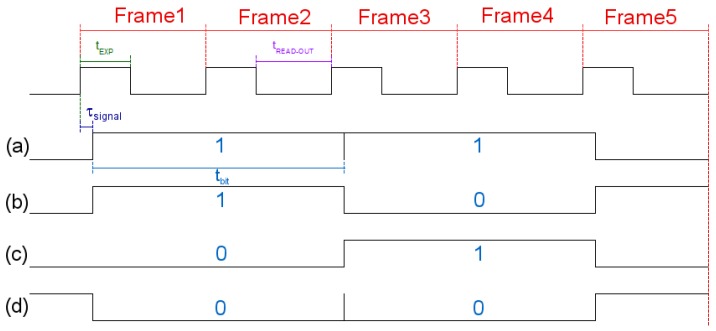
Possible transmitted signals. (**a**) Transmitting bit “1” after bit “1”, (**b**) Transmitting bit “0” after bit “1”, (**c**) Transmitting bit “1” after bit “0”, (**d**) Transmitting bit “0” after bit “0”.

At first, Equations (1) and (2) were used to determine the proportional separation distances between the emitters in function of the distance to the camera, the transmitter size and viewing angle, the desired pixels, and the dome camera characteristics. The difference in the height of the sensors was taken into account as an element for calculating the angle γ. A Monte Carlo simulation of 10^6^ cases was carried out for each projected distance (from 2.03 cm to 4.82 cm), and the worst case scenario (overlapping sensors) assuming the delay $\tau_{signal}$ of the transmitted signal as a uniformed distributed variable. The interference signal from the close-by LED matrix was turned on for all the cases. The user signal was created covering the four possible combinations of two consecutive bits as shown in Figure 18. In all the cases, the received bit was determined from the sum of the signal and the interference over the exposure time ($t_{exp}$) for the affected pixels in each channel (red, green and blue). The amplitude of the signal and the interference were calculated with the optical power of the LED matrix; and the Silicon responsivity and the Bayer filter response of the dome camera. The affected pixels were obtained from the NPSIR calculated in the previous experiment.

**Figure 19:**
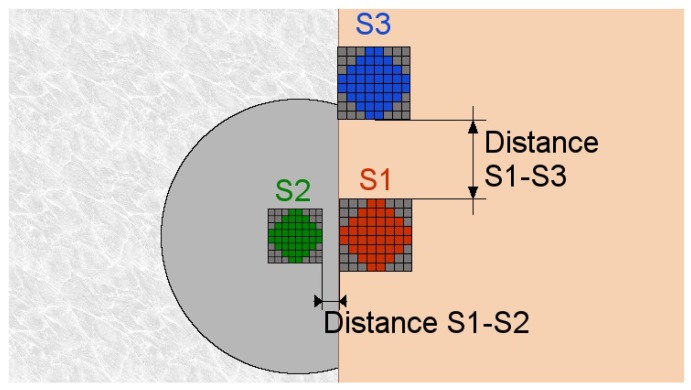
Dome camera perspective of the sensors.

Another simulation was also held. In this case, for each projected distance, the corresponding pixels SIR ($Pix_{S}/Pix_{I}$) was calculated using Equation (4) with the NPSIR values obtained in the previous experiment and the parameters of the simulated camera and emitters. Finally, the BER was calculated in function of the SIR. As shown in Figure 19, the sensors on the table (S1 and S3) had a wider disk representation than the sensor over the autonomous robot (S2) due to the height’s difference.

### Simulation Results

**Figure 20:**
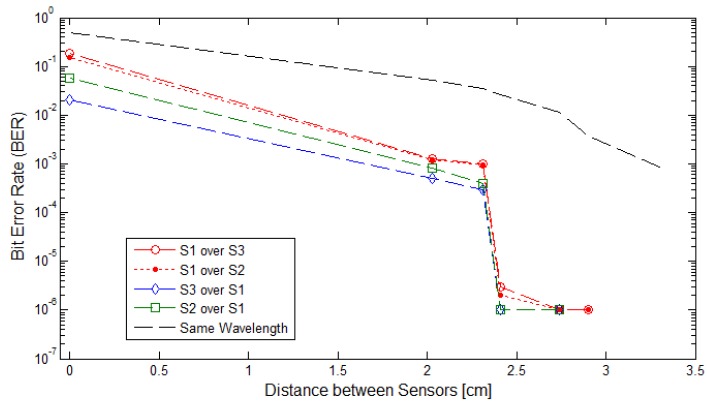
Monte Carlo Simulation. Bite Error Rate in function of the separation between sensors. The sensors transmitted On-Off Keying (OOK)-NRZ . signals in Red (S1), Green (S2) and Blue (S3).

The Monte Carlo simulations results, shown in Figure 20, have been divided into three categories:

- No distance between the LED matrices so the ROI is affected by the two overlapping sources equally,
- Distance between the LED matrices is greater than 2.00 cm and the two sources transmitted in the same wavelength, and
- Distance between the LED matrices is greater than 2.00 cm and the two sources transmitted with a different wavelength.

**Figure 21:**
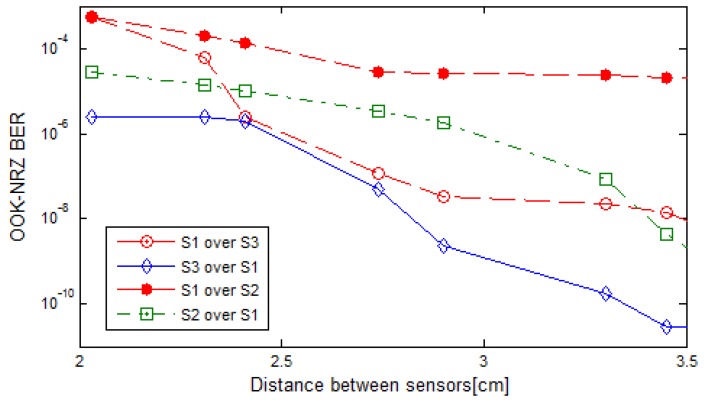
Normalized Power Signal to Interference Ratio (NPSIR) projection Simulation. Bite Error Rate in function of the separation between sensors. The sensors transmitted OOK-NRZ signals in Red (S1), Green (S2) and Blue (S3).

Results of the second wireless sensor network simulation are shown in Figure 21.

## 6. Conclusions

In this work, the spatial intersymbol interference from close-emitters within an OCC communication link was experimentally characterized in function of the transmitted wavelength, while the Normalized Power Signal to Interference Ratio (NPSIR) was introduced for measuring the chromatic interference independently of the chosen devices. Equations for easily determining the 2D pixel representation of real separation distances were deduced and validated.

The NPSIR of the pictures taken with the distance that assures perfect spatial separation of the sources represents minimum interference, as expected, in the majority of the cases greater than 40 dB. However, when the pictures have limited or critical spatial separation, the results depend greatly on the selected wavelength. For the transmissions in Blue (Figure 13) the minimum normalized interference came from close sources emitting in Red although this interference could affect the transmission. In the case of the LED transmitting in Green (Figure 14), close LEDs emitting in Blue and Red have similar influence over the communication link; but for transmissions in Red (Figure 15), there is no real interference from close emitters using Blue channel, but the communication could be affected by a LED sending messages in Green. The specific case of a device transmitting with white light (see Figure 16) presents almost the same interference from the different wavelength (Red, Blue, and Green) due to the fact that white light is produced by turning on the three LEDs at the same time.

In all cases, the most significant interference comes from possible close emitters transmitting in white or in the same channel of the target LED. Similar to channel allocation in RF systems, it is important to have close emitters in orthogonal channels: Red, Blue, Green.

The simulated scenario demonstrates the applicability of Equations (1) and (2) as an accurate tool for easily projecting distances into pixels under specific circumstances and the efficiency of Equation (4) for determining the interference independently of the system characteristics. Equations (1) and (2) were validated during the performance of the optical interference experiment with a mean error of less than one pixel.

The simulated scenario also shows that the deployment of this system is feasible. Using OOK-NRZ modulation there is no significant BER produced by close emitters when the sensors are separated more than 4.50 cm (zero for the Monte Carlo simulation and less than 2.85 × 10^−15^ for the NPSIR one), and the maximum BER of 1.3 × 10^−3^ from the Monte Carlo simulation (5.44 × 10^−4^ from the NPSIR simulation), obtained for 2.00cm separation, predicts that the communication link will work properly under real conditions.

The Monte Carlo simulation also demonstrated that the spatial intersymbol interference from close-emitters working in the same wavelength affects the system significantly. The use of the three channels (red, green and blue) reduce this interference, so future system designs should take advantage of the chromatic nature of the images and assign the different colors to the emitters in a similar way as WiFi allocates frequency bands.

Further research in this area is needed. The flickering problem will be addressed in future works, where the influence of external sources, such as solar radiation or illumination LEDs, should be taken into account. The presence of turbulence, particles and the temperature variation should also be characterized for outdoor environments.

In future works, Equations (1) and (2) could be used to determine the maximum range for specific OCC systems, assuring perfect segmentation of the different emitters while minimizing the interference from them. For example, assuming a Vehicular Network application where the headlights are independent emitters and the traffic cameras on the roads are the receivers, Equations (1) and (2) can be used to determine the maximum distance where a camera would be able to take photos from which specific emitter’s information can be extracted.

## Abbreviations

The following abbreviations are used in this manuscript:

ADC	Analog-to-Digital Converter
BER	Bit Error Rate
FOV	Field of View
FSO	Free-Space Optical
LOS	Line of Sight
MIMO	Multiple Input Multiple Output
NPSIR	Normalized Power Signal to Interference Ratio
NRZ	No Zero Return
OCC	Optical Camera Communication
OOK	On-Off Keying
ROI	Region of interest
SDMA	Space Division Multiple Access
SIR	Signal to Interference Ratio
SINR	Signal to Interference and Noise Ratio
SNR	Signal to Noise Ratio
VLC	Visible Light Communication
WSN	Wireless Sensor Networks
